# p5RHH nanoparticle-mediated delivery of AXL siRNA inhibits metastasis of ovarian and uterine cancer cells in mouse xenografts

**DOI:** 10.1038/s41598-019-41122-3

**Published:** 2019-03-18

**Authors:** Kathryn A. Mills, Jeanne M. Quinn, S. Tanner Roach, Marguerite Palisoul, Mai Nguyen, Hollie Noia, Lei Guo, Jawad Fazal, David G. Mutch, Samuel A. Wickline, Hua Pan, Katherine C. Fuh

**Affiliations:** 1 Center for Reproductive Health Sciences, Department of Obstetrics and Gynecology, Washington University School of Medicine, 425 S. Euclid Avenue, St. Louis, MO 63110 USA; 2 Division of Gynecologic Oncology, Department of Obstetrics and Gynecology, Washington University School of Medicine, 660 S. Euclid Avenue, St. Louis, MO 63110 USA; 3 Department of Cardiovascular Sciences, The USF Health Heart Institute, Morsani School of Medicine, University of South Florida, 4202 E. Fowler Avenue, Tampa, FL 33620 USA

## Abstract

Ovarian and uterine serous cancers are extremely lethal diseases that often present at an advanced stage. The late-stage diagnosis of these patients results in the metastasis of their cancers throughout the peritoneal cavity leading to death. Improving survival for these patients will require identifying therapeutic targets, strategies to target them, and means to deliver therapies to the tumors. One therapeutic target is the protein AXL, which has been shown to be involved in metastasis in both ovarian and uterine cancer. An effective way to target AXL is to silence its expression with small interfering RNA (siRNA). We investigate the ability of the novel siRNA delivery platform, p5RHH, to deliver anti-AXL siRNA (siAXL) to tumor cells both *in vitro* and *in vivo* as well as examine the phenotypic effects of this siRNA interference. First, we present *in vitro* assays showing p5RHH-siAXL treatment reduces invasion and migration ability of ovarian and uterine cancer cells. Second, we show p5RHH nanoparticles target to tumor cells *in vivo*. Finally, we demonstrate p5RHH-siAXL treatment reduces metastasis in a uterine cancer mouse xenograft model, without causing an obvious toxicity. Collectively, these findings suggest that this novel therapy shows promise in the treatment of ovarian and uterine cancer patients.

## Introduction

Ovarian cancer is the fifth leading cause of cancer death in women, and the five-year survival rate for ovarian cancer patients is less than 50%^1^. Because the symptoms of ovarian cancer (e.g., bloating, gas, nausea, and abdominal discomfort) are common with other diseases, most ovarian cancer patients present at an advanced stage^2^, at which point the cancer has already metastasized throughout the peritoneal space. Like ovarian cancer, high-grade uterine serous cancer is highly lethal, accounting for 40% of uterine cancer deaths^3,4^. Improving survival for ovarian and uterine cancer patients will require identifying therapeutic targets, strategies to target them, and means to deliver therapies to the tumors.

One candidate target protein involved in ovarian and uterine cancer metastasis is the TAM receptor tyrosine kinase family member AXL. Upon binding to its ligand, Gas6, AXL can activate several signal transduction pathways involved in cancer^5–11^. Recently, we found that high AXL expression in tumors correlates with poor survival in patients with high-grade uterine cancer. Additionally, AXL inhibition in uterine cancer cells decreased *in vitro* migration and invasion and *in vivo* metastatic potential in a mouse xenograft model^12^. AXL inhibition similarly prevents *in vitro* and *in vivo* invasion and migration of many other cancer types including brain, lung, and ovarian cancer^12–22^. Thus, targeting AXL could combat metastasis in both ovarian and uterine cancer.

One way to target AXL is to silence its expression with small interfering RNA (siRNA), and several nanoparticle systems have been developed to deliver siRNAs into cells. Although nanoparticles formed with cationic lipids and polymers effectively deliver siRNAs *in vivo*, these nanoparticle systems have not been used clinically because of potential toxicities caused by their propensity to aggregate with proteins in the serum^23–27^. An siRNA delivery system that can overcome these limitations is needed.

One promising nanoparticle siRNA delivery system, p5RHH^28^, was developed by selectively modifying a natural amphipathic cationic peptide: melittin^29–31^. Moreover, its c-terminal sequence has been altered with addition of multiple arginine and histidine residues for peptide-nucleotide interaction and pH sensing^28,32^. For the cytoplasmic siRNA delivery, p5RHH carries siRNA through peptide-nucleotide interaction enabled nanoparticle formation and the nanoparticles enter cells via endocytosis^28^. In the late endosome, p5RHH releases siRNA upon protonation of the histidine residues during the acidification of the endosome. Once p5RHH and siRNA are separated, the local high concentration of p5RHH in the endosome facilitates pore-formation in the endosomal membrane, allowing the siRNA to escape into the cytoplasm^28,32^. In the circulation, the siRNA-peptide complex is protected from destruction, and the peptide itself is at insufficient concentration to effect off-target membranolysis. Levels of administrated p5RHH is also noted to be well below the IC50, which it beween 200–400 μM. In the case of p5RHH, a trigger for pH-mediated particle disassembly with concurrent siRNA release is by endosomal acidification with concurrent siRNA release. The release of free p5RHH also leads to endosomal escape, which can only occur once concentrated in an endosome. This is a unique characteristic of p5RHH/siRNA nanoparticles to efficiently coordinate peptide and siRNA release within endosomal escape.

*In vitro*, p5RHH nanoparticles were used to deliver siRNAs to multiple cell types, including melanoma cells, endothelial cells, and macrophages for knockdown of different mRNAs, such as p65, p100, JNK2, and STAT3^32^. *In vivo*, p5RHH–siRNA nanoparticles were shown to decrease expression of the p65 subunit of NF-kappa-B in a mouse model of rheumatoid arthritis, leading to decreased ankle thickness and arthritic scores^33^. These nanoparticles were hypothesized to localize to arthritic joints by escaping from inflamed and leaky vasculature in the joints. A similar mechanism, the endothelial permeability and retention effect, allows nanoparticles to localize to cancer cells by escaping from inflamed and leaky vasculature in tumors. Given the ability of p5RHH to deliver siRNAs to tumor cells *in vitro* and to arthritic joints *in vivo*, as well as the similarities between arthritic joints and the tumor environment, we hypothesized that p5RHH could be used to deliver AXL-targeting siRNAs to high-grade ovarian and uterine serous tumor for potential clinical translation.

## Results

### p5RHH-siAXL nanoparticles decrease invasion and migration of uterine and ovarian cancer cells

**Figure 1:**
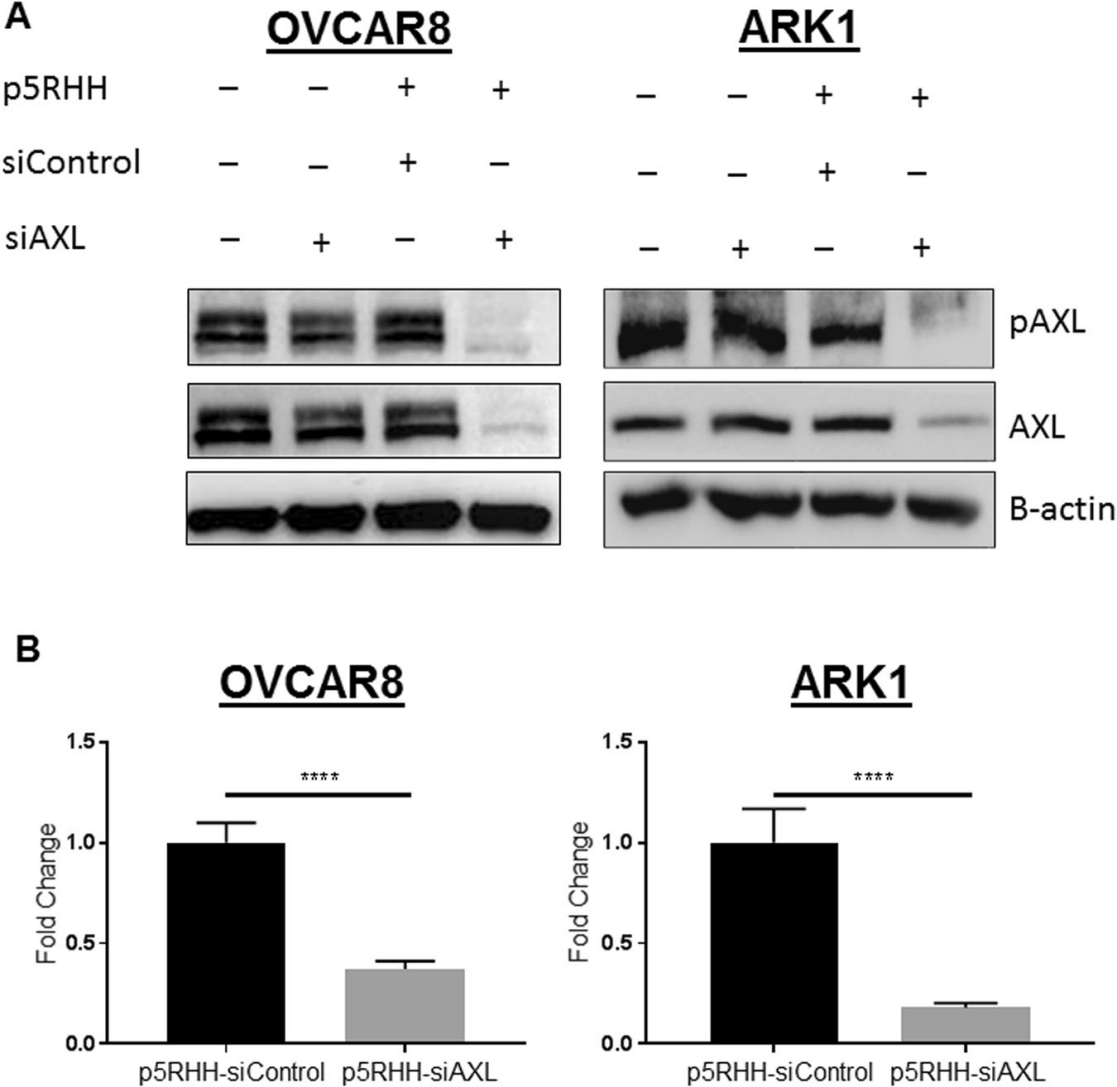
p5RHH nanoparticles effectively inhibit AXL and functional AXL. (**A**) Western blot analysis of phospho-AXL and AXL expression in ARK1 and OVCAR8 cell lines treated with vehicle, siAXL only, p5RHH-siControl, or p5RHH-siAXL. Actin is shown as a loading control. (**B**) qRT-PCR was performed on cells treated with p5RHH-siControl or p5RHH-siAXL and AXL mRNA expression was assessed. Data are represented as mean +/− SD and asterisks indicate significant decrease in expression compared to p5RHH-siControl as determined by the student’s t-test. ****P < 0.0001.

**Figure 2:**
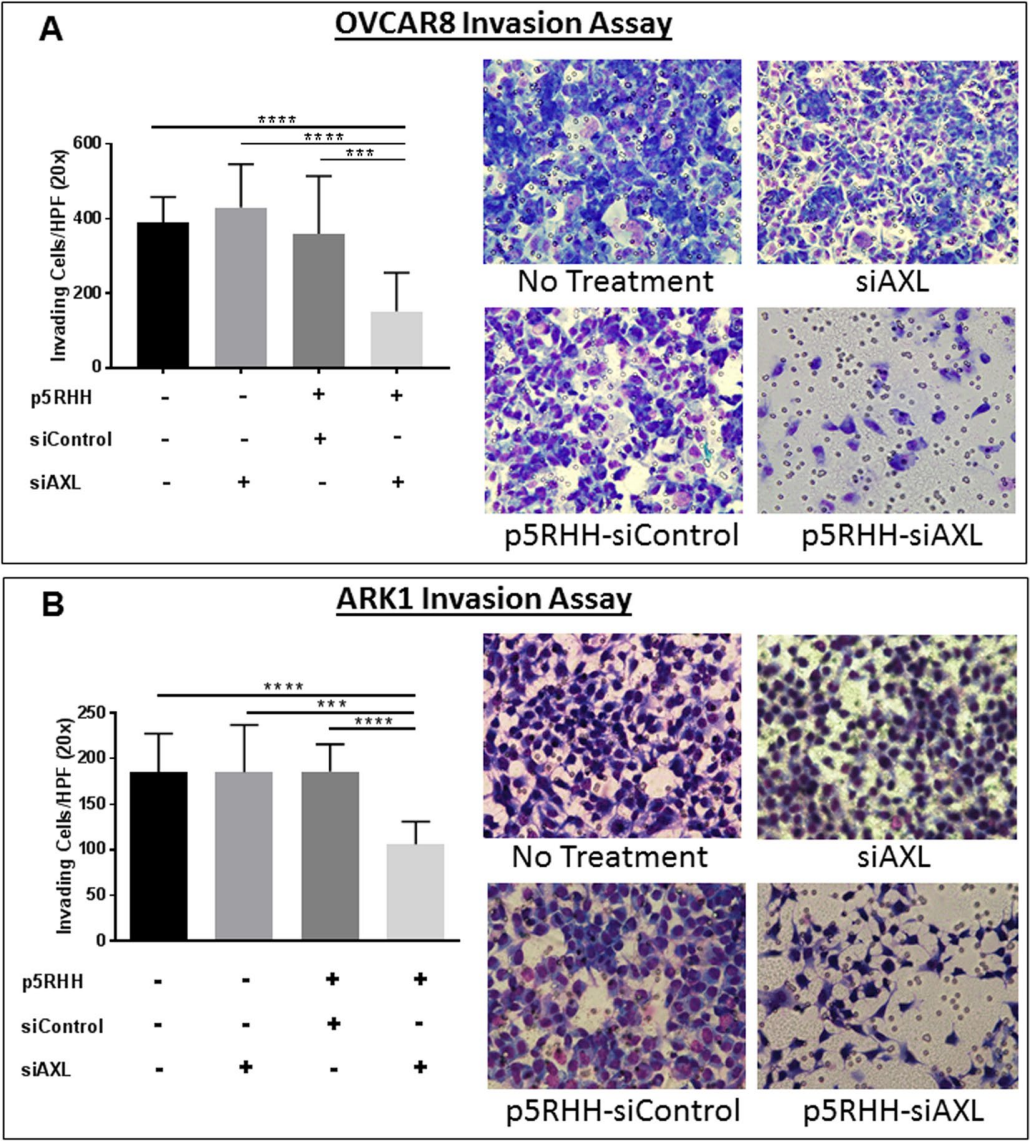
p5RHH-siAXL nanoparticles decrease *in vitro* invasion. Representative images of matrigel invasion assay of (**A**) OVCAR8 and (**B**) ARK1 cells treated with vehicle, siAXL, p5RHH-siControl, or p5RHH-siAXL. Graphs depict the number of invaded cells at 48 hours. Data are represented as mean +/− SD. ***P < 0.001, ****P < 0.0001 by student’s t-test.

To determine whether p5RHH nanoparticles could decrease migration and invasion, we first asked whether they could decrease AXL expression in uterine and ovarian serous cancer cells. We first confirmed knock down of AXL was maintained for as long as 96 hours after treatment with p5RHH-siAXL nanoparticles (Supplementary Fig. S5). We next treated ARK1 uterine serous cancer cells and OVCAR8 ovarian cancer cells with buffer, AXL siRNA (siAXL) alone, p5RHH-scrambled siRNA nanoparticles (p5RHH-siControl), or p5RHH-siAXL siRNA nanoparticles and evaluated protein expression after 72 hours. Treatment with p5RHH-siAXL siRNA nanoparticles led to a decrease in expression of AXL and phospho-AXL in both cell lines (Fig. 1A). Additionally, quantitative RT-PCR demonstrated that AXL RNA expression was significantly decreased by p5RHH-siAXL siRNA nanoparticles treatment in both cell lines (Fig. 1B). We next tested the p5RHH-siRNA-treated ARK1 and OVCAR8 cells in matrigel invasion and migration assays. In both cell lines, p5RHH-siAXL siRNA nanoparticles treated cells were significantly less invasive than those in the vehicle, siAXL only, and p5RHH-siControl nanoparticles treatment conditions (Fig. 2A,B). In addition, these assays were performed in ARK1 cells with constitutive short hairpin knockdown of AXL or a scrambled sequence. In cell lines with a scrambled sequence, treatment with p5RHH-siAXL particles resulted in even less invasion than cells harboring shAXL mutation (Supplementary Fig. S3A,B).

### p5RHH-siRNA nanoparticles localize to tumor cells *in vivo*

**Figure 3:**
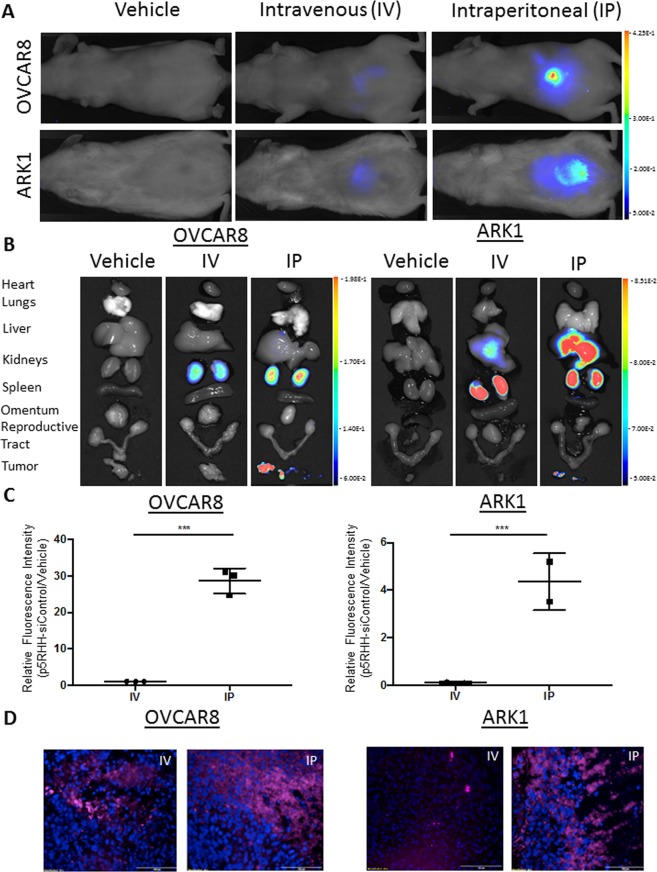
p5RHH-siControl-Quasar 705 nanoparticles localize to tumor cells in mouse xenograft models. Representative (**A**) *in vivo* and (**B**) *ex vivo* biodistribution images of IV- or IP-injected p5RHH-siControl-Quasar 705 probed nanoparticles in ARK1 and OVCAR8 tumor-bearing mice. (**C**) Quantitation of tumor fluorescence in mice injected with p5RHH-siControl-Quasar 705 relative to tumor fluorescence in mice injected with vehicle. (**D**) Representative images of ARK1 and OVCAR8 tumor cells in mice. Blue, nuclear dye DAPI; pink, Quasar 705 fluorescence. Data are represented as mean +/− SD. ***P < 0.001 by unpaired t-test.

To assess the *in vivo* utility of p5RHH-siAXL nanoparticles, we first wanted to determine whether or not p5RHH would localize to tumors as it did to inflamed joints in the mouse model of arthritis^33^, and we wanted to identify the best delivery method. To answer these questions, we injected ARK1 and OVCAR8 cells into NOD/SCID and NU/FOX mice, respectively. After tumors had established, we injected mice either intraperitoneally (IP) or intravenously (IV) with p5RHH-siControl nanoparticles, in which siControl siRNA was covalently conjugated with the fluorescent probe Quasar 705. Fluorescent images taken 24 hours later revealed that for both tumor cell types, the IP-injected mice had more fluorescent signal in the peritoneal cavity than did the IV-injected mice (Fig. 3A). In addition, *ex vivo* imaging of tumors of both types revealed that a greater quantity of IP-injected nanoparticles compared to IV-injected nanoparticles localized to tumors (Fig. 3B,C). Finally, we found that, following both injection routes, the fluorescent probe was localized intracellularly in tumors (Fig. 3D), and the tumors from the IP-injected mice had more intracellular fluorescence than those from IV-injected mice. Thus, we conclude that the p5RHH nanoparticles localize to and release their contents into tumor cells and that IP administration is more effective than IV administration. Moreover, in this specific pathological condition, it is feasible for IP-administration.

### p5RHH-siAXL treatment significantly reduces *in vivo* metastasis and is non-toxic

**Figure 4:**
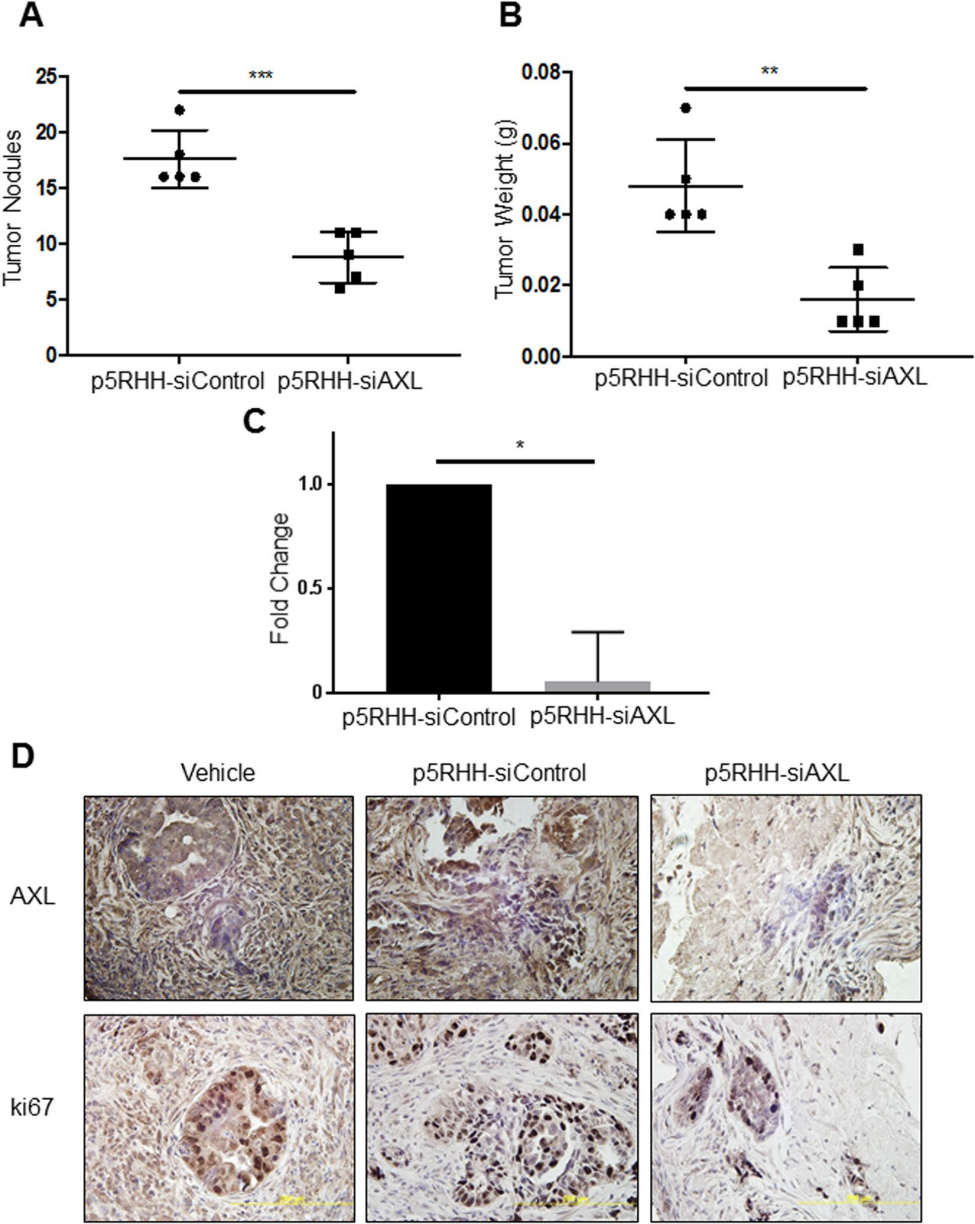
Treatment with p5RHH-siAXL significantly reduces metastasis of ARK1 xenografts. Graphs depicting the (**A**) number of peritoneal tumor nodules and (**B**) total tumor weight per mouse treated with either p5RHH-siControl (n = 5) or p5RHH-siAXL (n = 5). (**C**) qRT-PCR was performed on tumors treated with p5RHH-siControl or p5RHH-siAXL and AXL mRNA expression was assessed. (**D**) Representative images of AXL and ki67 immunohistochemistry of tumor samples from vehicle, p5RHH-siControl, or p5RHH-siAXL mice at 40X magnification. Data are represented as mean +/− SD. *P < 0.05, **P < 0.01, ***P < 0.001 by unpaired t-test.

Given that p5RHH-siRNA nanoparticles could localize to tumors and that p5RHH-siAXL treatment *in vitro* decreases invasion and migration, we investigated whether p5RHH-siAXL treatment could reduce *in vivo* metastasis of ARK1 cells. After two weeks of treatment, mice that received p5RHH-siAXL nanoparticle treatment had significantly fewer intraperitoneal tumor nodules (8.8 vs. 17.6) and lower overall tumor mass (0.016 g vs. 0.048 g) than mice that received p5RHH-siControl nanoparticles (Fig. 4A,B). We found a similarly lower number of intraperitoneal OVCAR8 tumor nodules in mice that received p5RHH-siAXL nanoparticles than in those that received p5RHH-siControl nanoparticles (Supplementary Fig. S1). In addition, quantitative RT-PCR demonstrated that AXL RNA expression was significantly decreased in tumors treated with p5RHH-siAXL nanoparticles compared to p5RHH-siControl nanoparticle treated tumors (Fig. 4C). In addition, tumors from mice that received p5RHH-siAXL had decreased levels of AXL IHC expression compared to p5RHH-siControl and vehicle mice (Fig. 4D). In addition, analysis of mouse tumors showed significantly decreased markers of metastasis (Matrix metallopeptidase 2 and Matrix metallopeptidase 3) in tumors from mice treated with p5RHH-siAXL nanoparticles compared to control (Supplementary Fig. S4). Together, these results show p5RHH-siAXL treatment reduces *in vivo* tumor metastasis.

**Figure 5:**
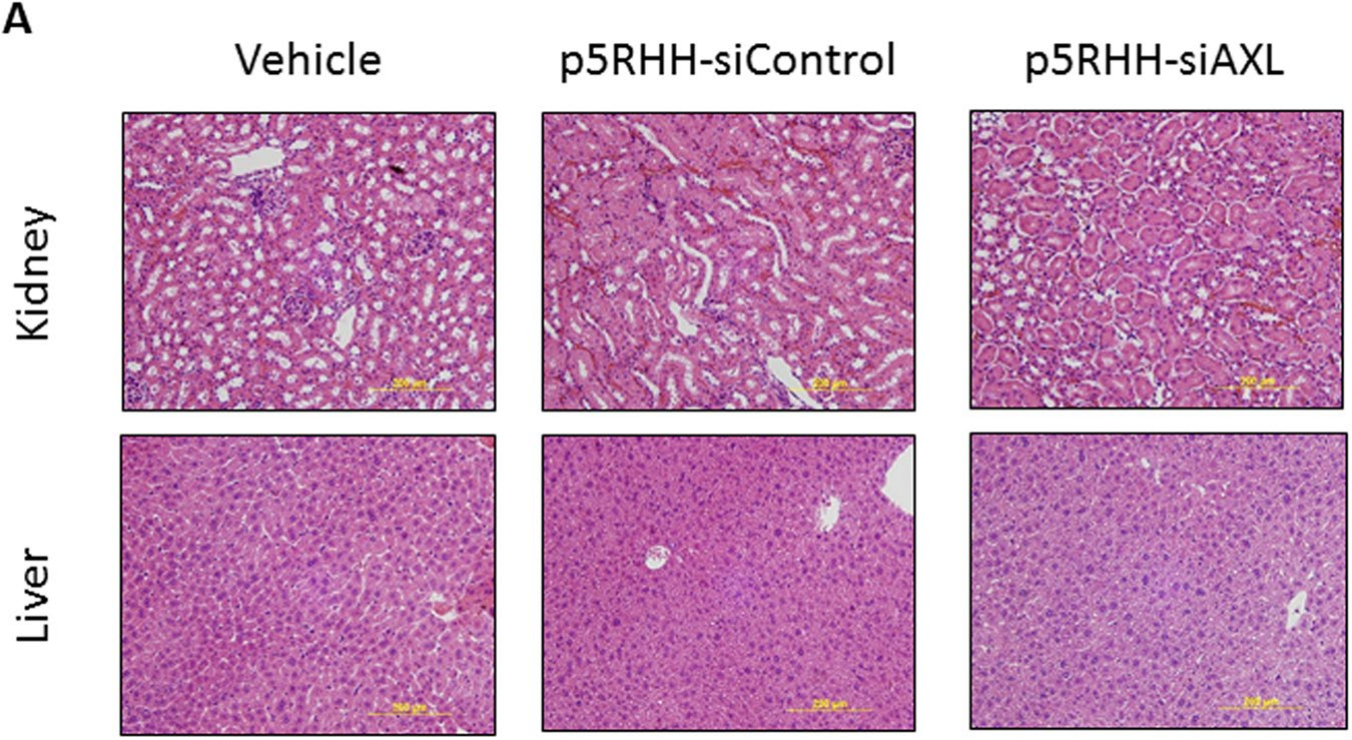
Histological evaluation of major organs. (**A**) Representative images of hematoxylin and eosin-stained kidney and liver from OVCAR8 xenograft-bearing mice.

To determine whether p5RHH-siAXL nanoparticle treatment caused any hematologic toxicity, we performed blood analyses on samples collected from the mice. We detected no significant difference in white blood cell, red blood cell, hemoglobin, or platelet counts between buffer-, p5RHH-siControl nanoparticle-, and p5RHH-siAXL nanoparticle-treated mice bearing ARK1 or OVCAR8 cells (Table 1). Furthermore, pathologic analysis of major organs revealed no histological differences between the treatment groups (Fig. 5A). Together, these findings indicate that p5RHH-siAXL nanoparticle treatment has preferable safety profile.

**Table 1:** p5RHH-siAXL complete blood count.

	ARK1				OVCAR8			
	Vehicle	p5RHH-siControl	p5RHH-siAXL	Normal Range	Vehicle	p5RHH-siControl	p5RHH-siAXL	Normal Range
WBC (10^3/uL)	3.2	1.6 ± 0.1	5.0 ± 3.1	0.96–4.68	4.6 ± 1.3	1.7 ± 0.8	6.3 ± 1.8	1.42–10.25
RBC (10^6/uL)	8.1	8.6 ± 0.3	8.7 ± 0.1	8.21–10.48	9.4 ± 0.1	10.3 ± 0.1	9.7 ± 0.1	6.82–10.53
HGB (g/dL)	13.1	12.8 ± 0.5	13.0 ± 0.1	12.1–17.6	14.4 ± 0.2	15.1 ± 0.6	14.0 ± 0.1	10.9–15.9
PLAT (10^3/uL)	830	1233.5 ± 12.5	1048 ± 178	651–1878	859.5 ± 58.5	949.5 ± 166.5	723.5 ± 31.5	376–1796

*There were no difference between means found by one-way ANOVA analysis.

Complete blood count table of blood samples from ARK1 and OVCAR8 mice treated with vehicle, p5RHH-siControl, or p5RHH-siAXL. Data are represented as mean +/− SD and significance was determined by one-way ANOVA.

## Discussion

High-grade serous ovarian and uterine cancers are extremely lethal^34,35^. Although many patients respond to initial chemotherapy, most will eventually relapse and die because of metastatic disease^3,4^. Recently, AXL was shown to have a role in ovarian and uterine cancer metastasis, making it a therapeutic target to combat metastasis in these cancers. Here, we present evidence that p5RHH-siAXL nanoparticles inhibit AXL expression both *in vitro* and *in vivo* in ovarian and uterine cancer models. Additionally, in *in vitro* assays, p5RHH-siAXL nanoparticle treatment reduced invasion and migration ability of ovarian and uterine cancer cells. Finally, p5RHH-siAXL nanoparticles target to tumor cells and reduce metastasis without causing any obvious toxicity. Additionally, we focused on p5RHH-siControl and p5RHH-siAXL rather than p5RHH alone for toxicity given that p5RHH is only released once inside the endosome. Thus, there is no activity of p5RHH alone unless inside the endosome^36^. p5RHH exists in nanoparticle form only and is only released in free form once inside the endosome. Without siRNA components, there would be no nanoparticle and the siRNA would be subject to immediate effects of RNAases in circulation and the peptide rapidly cleared by the kidney, and thus p5RHH alone exerts no therapeutic activity. The peptide itself, being bound in the nanoparticle, does not circulate freely as it requires a low pH (<5) to cause the nanoparticle to disaggregate^28^. Such conditions exist inside the endosome as previously published. Accordingly, there is no opportunity for p5RHH to exist in free form and cause toxicity. Once the nanoparticles are disaggregated in the endosome upon lowering of pH, the peptide is released causing endosomolysis. The peptide, now in free form, exerts no toxicity on any tissues or organs^33,37,38^.

In previous work, we showed that silencing AXL with short hairpin RNA decreased migration and invasion *in vitro* and reduced *in vivo* metastatic potential of uterine cancer^12^. However, short hairpin RNAs must be delivered via a viral vector, which can integrate into DNA and increase the risk of new mutations^39^. In contrast, p5RHH nanoparticles show promise as a clinically translatable option for safely silencing gene expression.

Our data indicate that p5RHH-siAXL safely targets tumors without causing off-target toxicities. The full body fluorescent images show that the nanoparticles were limited to the peritoneal cavity regardless of administration route (IV or IP) and did not accumulate in any upper respiratory organs. The only major organs to show fluorescence signal in the *ex vivo* images were the kidney and liver. The renal fluorescence was expected because p5RHH is excreted through the renal system. Although fluorescence signal was present in the liver, pathologic analysis revealed no signs of tissue damage. Additionally, the hematologic data demonstrate no difference among the treatment groups. Collectively, these findings suggest that this therapy shows promise in the treatment of ovarian and uterine cancer patients.

## Methods

### Cell Lines and Conditions

The uterine serous cancer cell line ARK1 was provided by Shi-Wen Jiang (Mercer University School of Medicine, Savannah, GA, USA), and the high grade serous ovarian cancer cell line OVCAR8 was purchased from the National Cancer Institute-Frederick DCTD tumor cell line repository. Cells were maintained in RPMI (Sigma, St. Louis, MO) supplemented with 10% heat-inactivated fetal bovine serum (FBS) (Sigma, St. Louis, MO) and 1% penicillin and streptomycin (Invitrogen, Carlsbad, CA) at 37 °C in a 5% CO_2_ incubator. For p5RHH treatment, cells were incubated in FBS-containing media without antibiotics for one day, then incubated in FBS-free, antibiotic-free media for 6 hours during incubation with p5RHH nanoparticles.

### Preparation of p5RHH nanoparticles

p5RHH peptides were prepared and stored as previously described^32^. For *in vitro* use, p5RHH-siRNA nanoparticles were constructed by mixing 1 µL of 20 mM p5RHH peptide with 10 µL of 20 µM siRNA in 389 µL of serum-free, antibiotic-free RPMI (Sigma, St. Louis, MO). The mixture was incubated at 37 °C for 40 minutes before use. Human AXL siRNA (ON-TARGETplus SMART pool #L-003104-00-0005) and siControl (ON-TARGETplus Non-targeting pool #D-001810-10-05) (Dharmacon, Lafayette, CO) were dissolved at 20 μM in molecular grade water (Mediatech, Manassas, VA) and stored at −20 °C before use.

For *in vivo* use, p5RHH-siRNA nanoparticles were constructed by mixing 4 µl of 100 mM p5RHH peptide with 8 µL of 100 µM siRNA in 188 µl of phosphate buffered saline (PBS) with Ca^2+^ and Mg^2+^ (Sigma, St. Louis, MO) per mouse. The mixture was then incubated on ice for 8 minutes and mixed with 0.8 µl of 0.2 g/ml mouse albumin (Sigma, St. Louis, MO) reconstituted in molecular grade water. Human AXL siRNA (Sense: 5′–ACAGCGAGAUUUAUGACUAUU; 5′-GGUA CCGGCUGGCGUAUCAUU; 5′-GACGAAAUCCUCUAUGUCAUU; 5′-GAAGGAGACCCGUUAUGGAUU) for *in vivo* use was created as a custom product (Dharmacon, Lafayette, CO) of four ON-TARTGET plus sequences against AXL reconstituted to 100 µM with 1x siRNA Buffer then pooled. *In Vivo* siControl (Dharmacon, Lafayette, CO) was reconstituted to 100 µM in 1x siRNA Buffer.

### Preparation of p5RHH-siSCRM-Quasar 705

Nanoparticle complexes were prepared by combining p5RHH and eGFP-Quasar 705-probed-siControl at 100:1 (Sigma, St. Louis, MO) in PBS supplemented with Ca^2+^ and Mg^2+^ (Sigma, St. Louis, MO). The mixture was incubated at 37 °C for 40 minutes.

### p5RHH-siRNA Treatment

Twenty-four hours before transfection, ARK1 or OVCAR8 cells were plated in RPMI medium supplemented with 10% FBS without antibiotics. The nanoparticle solution was then added and cells were starved in antibiotic-free, FBS-free media. After 6 hours, cells were placed in RPMI with 10% heat inactivated FBS.

### Western Blot Analysis

After 72 hours of exposure to specified treatment, cells were lysed in 9 M Urea, 0.075 M Tris, pH 7.6. Protein concentration was determined by using the Bradford assay, and lysates were subjected to reducing SDS/PAGE by standard methods. Western blots were incubated with primary antibodies against AXL (R&D Systems; 1:1000) and phospho-AXL (Cell Signaling; 1:500). To confirm equal protein loading, blots were probed with antibodies specific for β-actin (Sigma Aldrich; 1:3000). Horse-radish peroxidase-conjugated secondary antibodies were used to detect protein expression (Jackson ImmunoResearch, West Grove, PA) and chemiluminescence was measured on a ChemiDoc (Bio-Rad Laboratories).

### cDNA Preparation and qPCR

After 72 hours of indicated treatment, total RNA was isolated from ARK1 and OVCAR8 by using the RNeasy Mini Kit (Qiagen). cDNA was made from 1 µg of RNA by using the SuperScript IV system (Thermo Fisher Scientific) according to the manufacturer’s directions. Applied Biosystems 7500 detection system and SYBR-green master mix (Thermo Fisher Scientific) were used to perform qPCR. mRNA expression was normalized to 18 S ribosomal RNA, and fold change was calculated by using the 2^−ΔΔ^*C*_t_ method. Primer sequences for AXL were published previously^17,40,41^.

### Transwell Invasion Assay

Cells were treated with p5RHH-siRNA for 48 hours, starved for 24 hours in RPMI with 1% FBS, and then used for invasion assays. The matrigel invasion assay was performed in Boyden transwell chamber according to the manufacturer’s protocol with matrigel (Corning) diluted to 0.5 mg/ml in PBS. Chemoattractant made of 20% FBS, 0.4 µL of 100 ng/mL GAS6 (R&D systems), and 5 µl of 1 mg/ml human fibronectin (Corning) was added to the bottom well. Cells were allowed 48 hours to invade. Invaded cells were fixed, stained, and counted in four different fields at 20x magnification. All experiments were performed in triplicate.

### Immunohistochemistry

Major organs from mice were resected, fixed in fresh formalin for 48 hours, subjected to 10-minute washes in increasing ethanol concentration up to 70% ethanol, and then processed for paraffin embedding. Slides were deparaffinized in Xylene, gradually rehydrated in decreasing concentrations of ethanol followed by heat-induced antigen retrieval in sodium citrate, pH 6, for 8 minutes. Slides were probed with polyclonal goat anti-Human AXL antibody (R&D Systems; 1:40) at 4 °C overnight. Slides were then washed and incubated with HRP-conjugated secondary antibody (Vector Laboratories; 1:200). Antibody complexes were detected with 3,3′ – diaminobenzidine (Dakos), and slides were counter stained with Mayer’s Hematoxylin. Sections (5 µm) were mounted on glass microscope slides. Tissues from major organs were stained with hematoxylin and eosin (H&E) to identify any effects from treatment.

### Murine Xenograft Studies

All procedures involving animals and their care were performed in accordance with the guidelines of the American Association for Accreditation for Laboratory Animal Care and the U.S. Public Health Service Policy on Humane Care and Use of Laboratory Animals. All animal studies were also approved and supervised by the Washington University Institutional Animal Care and Use Committee in accordance with the Animal Welfare Act, the Guide for the Care and Use of Laboratory Animals, and NIH guidelines.

Cells were prepared in 0.2 ml of MgCl_2_ and CaCl_2_ supplemented PBS (Sigma), and 1 × 10^7^ ARK1 and 5 × 10^6^ OVCAR8 cells were injected intraperitoneally into female 6–8-week-old NOD SCID (Jackson Laboratory) or NU/FOX (Charles River) mice, respectively. Mice were monitored for adverse events until sacrifice. Tumor samples were fixed in formalin and embedded in paraffin or snap frozen for further analysis.

### Nanoparticle Localization Studies

ARK1 and OVCAR8 cells were injected into mice and allowed 36 and 28 days, respectively, to establish tumors. Mice were fed Irradiated AIN-93M chow (Research Diets, New Brunswick, NJ). Vehicle control mice were injected with PBS supplemented with MgCl_2_ and CaCl_2_ both intraperitoneally and intravenously, and the treatment groups were injected either intraperitoneally or intravenously with p5RHH-eGFP-Quasar 705-probed-siControl nanoparticles. After 24 hours, the mice were imaged with the Pearl Trilogy Small Animal Imaging System (LI-COR Biosciences, Lincoln, NB). During *in vivo* imaging, mice were maintained under isoflurane inhalation anesthesia until sacrifice by both CO_2_ euthanasia and cervical dislocation. Before dissection and *ex vivo* tissue imaging mice were systemically perfused with PBS. Image acquisition settings were: excitation 685 nm, emission 720 nm, field of view 11.2 cm × 8.4 cm, exposure time 2 sec. For microscopic localization, tumor OCT blocks were cryosectioned (10 µm), mounted in mounting media (Cat#: H-1200, Vector Laboratories, Burlingame, CA), and imaged with an Olympus BX63 fluorescence microscope (Olympus, Center Valley, PA). All images were obtained at the same imaging acquisition conditions.

### *In vivo* nanoparticle-mediated siRNA delivery

ARK1 and OVCAR8 cells were injected into mice and allowed to establish tumors for 6 days and 3 days, respectively. ARK1 xenograft mice models were injected intraperitoneally with p5RHH-siControl or p5RHH-siAXL. OVCAR8 xenograft models were injected intraperitoneally with either PBS, p5RHH-siControl, or p5RHH-siAXL. The p5RHH-siRNA intraperitoneal dosing regimen was determined by treating mice with increasing ratios of p5RHH to siAXL with a final ratio of 100:1. Final therapeutic dosing cycles were 3 days of treatment followed by 1 day off for a span of 2 weeks. Mice were then sacrificed and dissected.

### Statistical analyses

Graphpad Prism 7.03 software was used for statistical analyses. Two-tailed unpaired Student’s t tests and one-way ANOVA were performed to analyze statistical differences between groups. *P* < 0.05 was considered statistically significant.

## Supplementary information

Supplementary information
