# Supplementary material for: p5RHH nanoparticle-mediated delivery of AXL siRNA inhibits metastasis of ovarian and uterine cancer cells in mouse xenografts
Source: Sci Rep. 2019 Mar 18;9:4762. doi: 10.1038/s41598-019-41122-3 (PMC6423014; doi:10.1038/s41598-019-41122-3)
Supplement: Supplementary file 1 — Supplementary information [file 41598_2019_41122_MOESM1_ESM.pdf]

p5RHH nanoparticle-mediated delivery of AXL siRNA inhibits metastasis of ovarian and uterine cancer cells in mouse xenografts

Kathryn A. Mills[1,2,+], Jeanne M. Quinn[1,+], S. Tanner Roach[1], Marguerite Palisoul[1,2], Mai Nguyen[1], Hollie Noia[1], Lei Guo[1], Jawad Fazal[3], David G. Mutch[2], Samuel A. Wickline[3], Hua Pan[3,*], Katherine C. Fuh[1,2,*]

[1]Center for Reproductive Health Sciences, Department of Obstetrics and Gynecology, Washington University School of Medicine, 425 S. Euclid Avenue, St. Louis, MO 63110

[2]Division of Gynecologic Oncology, Department of Obstetrics and Gynecology, Washington University School of Medicine, 660 S. Euclid Avenue, St. Louis, MO 63110

[3]Department of Cardiovascular Sciences, The USF Health Heart Institute, Morsani School of Medicine, University of South Florida, 4202 E. Fowler Avenue, Tampa, FL 33620, USA

A

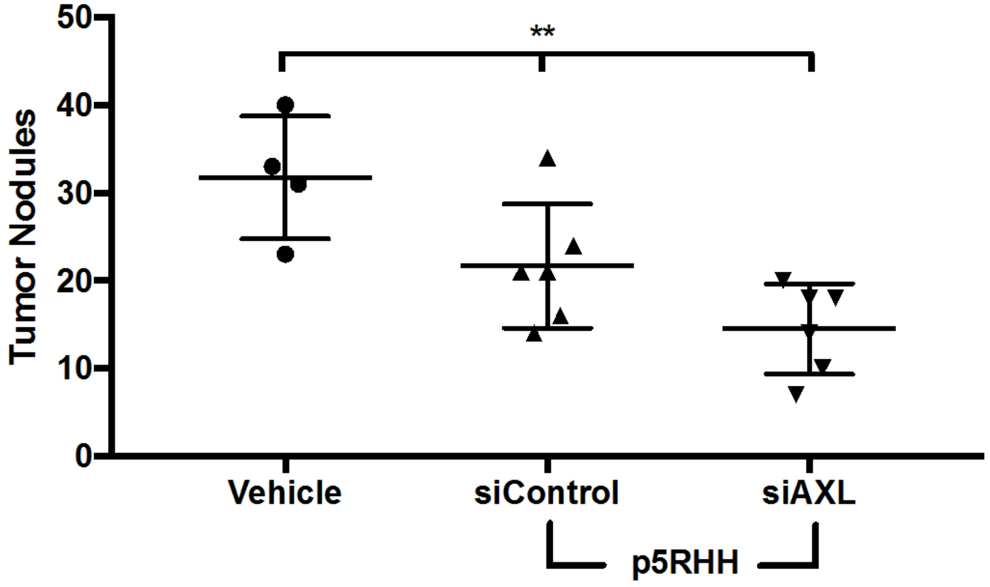

Supplementary Figure S1. Treatment with p5RHH-siAXL reduces OVCAR8 tumor nodules in mouse xenografts. A) Graph depicts the number of peritoneal tumor nodules per mouse treated with vehicle (n=4), p5RHH-siControl (n=6), or p5RHH-siAXL (n=6). Data are presented as mean +/- SD. $**P < 0.01$ by one-way Anova.

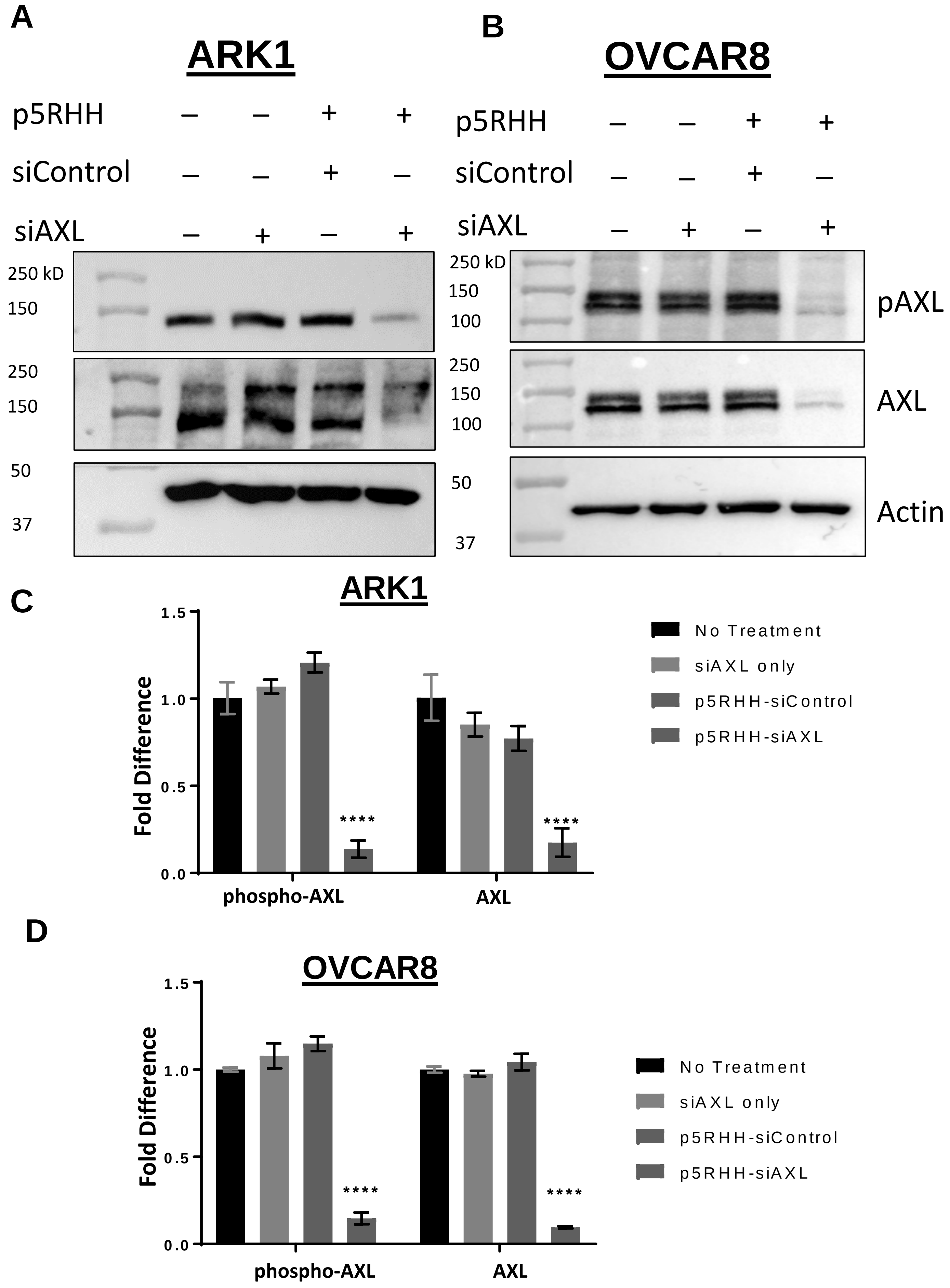
A
ARK1
p5RHH – – + +
siControl – – + –
siAXL – + – +
250 kD
150
250
150
50
37
B
OVCAR8
p5RHH – – + +
siControl – – + –
siAXL – + – +
250 kD
150
100
250
150
100
50
37
pAXL
AXL
Actin
C
ARK1
1.5
1.0
0.5
0.0
Fold Difference
****
****
phospho-AXL
AXL
No Treatment
siAXL only
p5RHH-siControl
p5RHH-siAXL
D
OVCAR8
1.5
1.0
0.5
0.0
Fold Difference
****
****
phospho-AXL
AXL
No Treatment
siAXL only
p5RHH-siControl
p5RHH-siAXL

Supplementary Figure S2. A,B) Full length blots of Figure 1A. The red lines indicated the cropping locations. **C,D)** Densitometric analysis of AXL and phospho-AXL expression for blots in Figure 1A

A

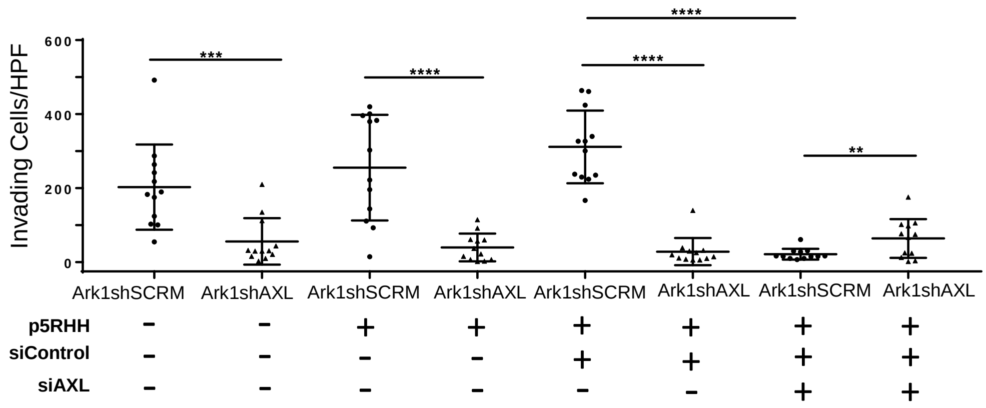
Invading Cells/HPF
600
400
200
0
***
****
****
****
**
Ark1shSCRM
Ark1shAXL
Ark1shSCRM
Ark1shAXL
Ark1shSCRM
Ark1shAXL
Ark1shSCRM
Ark1shAXL
p5RHH - - + + + + + +
siControl - - - - + + + +
siAXL - - - - - - + +

B

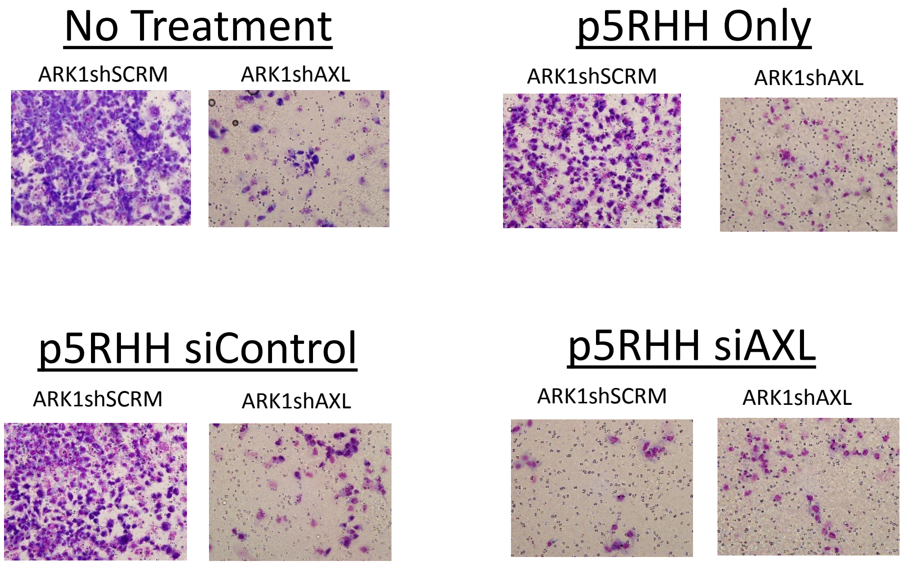
No Treatment
ARK1shSCRM
ARK1shAXL
p5RHH Only
ARK1shSCRM
ARK1shAXL
p5RHH siControl
ARK1shSCRM
ARK1shAXL
p5RHH siAXL
ARK1shSCRM
ARK1shAXL

Supplementary Figure S3. Ark1shSCRM cell line have reduced invasion after treatment of p5RHH-siAXL in vitro A) Matrigel Invasion results comparing ark1shSCRM and ark1shAXL no treatment, treatment with p5RHH only, treatment with p5rHH-siControl, and p5rHH-siAXL. Significance was calculated using one-way ANOVA. ** $P < 0.01$; *** $P<0.001$; **** $P < 0.0001$. **B)** Representative images of matrigel invasion, 20x magnification

A

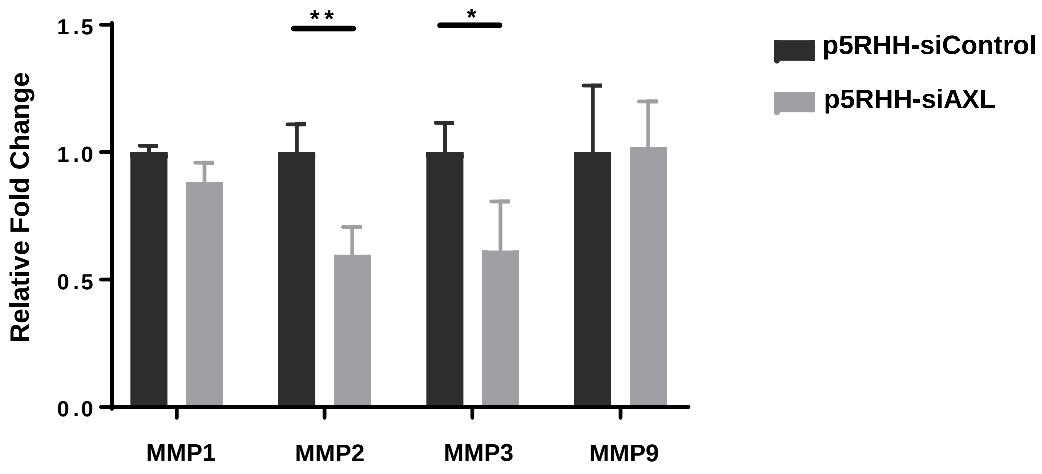

Relative Fold Change
1.5
1.0
0.5
0.0
**
*
MMP1
MMP2
MMP3
MMP9
p5RHH-siControl
p5RHH-siAXL

- **Supplementary Figure S4.** qPCR was performed on FFPE tumors treated with p5RHH-siControl or p5RHH-siAXL and phospho-AXL and AXL mRNA expression was assessed. Significance was calculated using SD or DDC_T. Error bars indicate the range of fold change. * $P < 0.05$; ** $P < 0.01$.

A

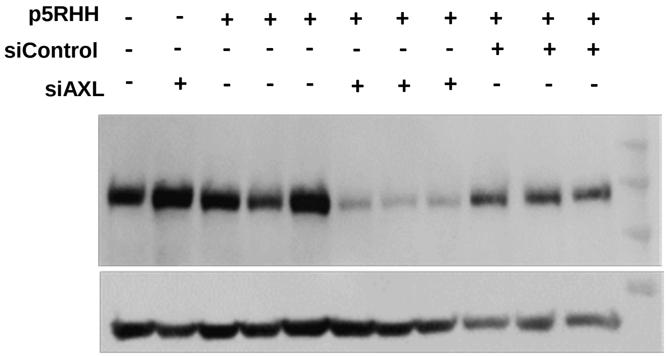

B

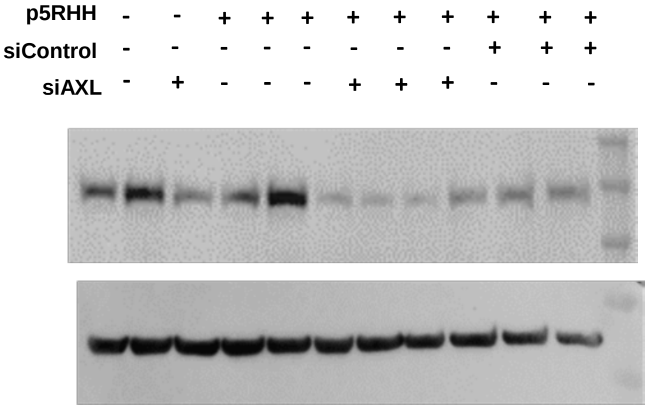

Supplementary Figure S5. A) Western blot analysis of AXL at 48 hours in Ark1 cells untreated and treated with siAXL alone, p5RHH alone, p5RHH-siAXL, and p5RHH-siControl. **B)** Western blot analysis of AXL at 96 hours in Ark1 cells treated as stated in A.
